# Bactericidal and anti-quorum sensing activity of repurposing drug Visomitin against *Staphylococcus aureus*

**DOI:** 10.1080/21505594.2024.2415952

**Published:** 2024-10-10

**Authors:** Ruolan Wu, Yuan Wu, Pingyun Wu, Huilong Li, Pengfei She

**Affiliations:** Department of Laboratory Medicine, The Third Xiangya Hospital of Central South University, Changsha, China

**Keywords:** Methicillin-resistant *Staphylococcus aureus*, antimicrobial, Visomitin, quorum sensing, biofilm, haemolysis

## Abstract

With the growing antibiotic resistance in *Staphylococcus aureus*, it is imperative to develop innovative therapeutic strategies against new targets to reduce selective survival pressures and incidence of resistance. In *S. aureus*, interbacterial communication relies on a quorum sensing system that regulates gene expression and physiological activities. Here, we identified that Visomitin, an antioxidant small molecule, exhibited bactericidal efficacy against methicillin-resistant *S. aureus* and its high tolerance phenotypes like intracellular bacteria and persister cells without inducing resistance. Critically, sub-minimal inhibitory concentrations (sub-MICs) of Visomitin could serve as a potent quorum-quencher reducing virulence production (such as haemolysin and staphyloxanthin), along with inhibiting biofilm formation, self-aggregation, and colony spreading of *S. aureus*. These effects were probably mediated by interfering with the *S. aureus* accessory gene regulator quorum sensing system. In summary, our findings suggest that Visomitin shows dual antimicrobial effects, including bactericidal effects at the concentrations above MIC and quorum sensing inhibition effects at sub-MICs, which holds promise for treating MRSA-related refractory infections.

## GRAPHICAL ABSTRACT

**Figure:**
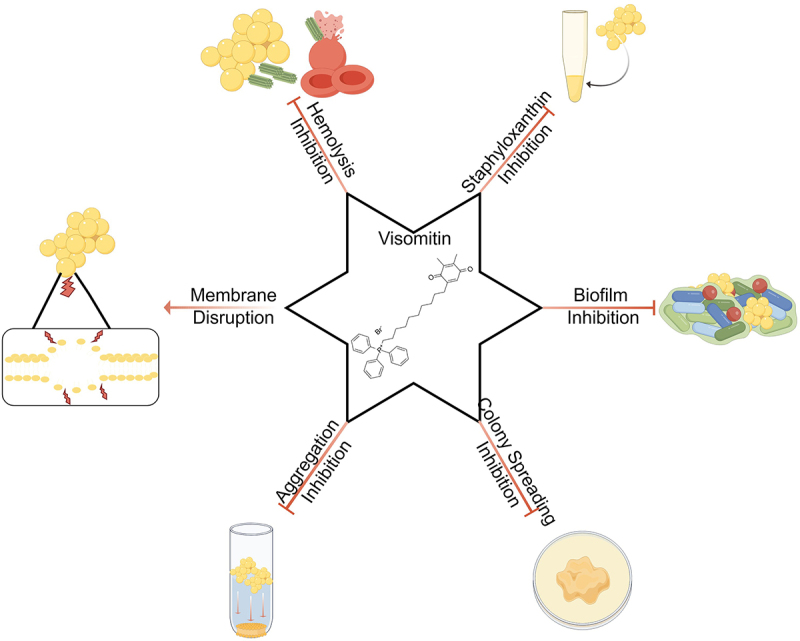
By Figdraw.

## Introduction

*Staphylococcus aureus*, a common Gram-positive pathogen, provokes diverse diseases varying from mild skin infection to fatal diseases such as pneumonia and endocarditis [1,2]. Antibiotics, including penicillin and methicillin, have been utilized to cure *S. aureus*-related infections [3]. Unfortunately, the abuse of antibiotics has largely increased drug-resistant bacteria, resulting in refractory infections [4,5]. According to the China Antimicrobial Surveillance Network, in 2022, Methicillin-resistant *S. aureus* (MRSA) accounted for 30.4% of the clinically isolated *S. aureus* [6]. The occurrence of MRSA, as well as the mortality rate of infected patients, has continued to increase in other countries [4,7]. More seriously, vancomycin-resistant *S. aureus* has been identified in clinical infections, posing a significant challenge to human health [8].

The pathogenic factors generated by *S. aureus* are diverse. Planktonic cells generally cause acute infections by secreting toxins and exoenzymes [9], while chronic *S. aureus* infections are frequently linked to biofilms, persister cells, and quorum sensing [10,11]. Quorum sensing refers to the ability of microorganisms to sense bacterial cell density and respond via gene expression regulation to adapt to the changing environmental conditions [12,13]. It could modulate a wide range of microbial behaviours, including nutrient acquisition, immune evasion, biofilm formation, virulence production, and motility, all of which largely promote the *S. aureus* pathogenicity [14]. Since quorum-sensing inhibitors aim to attenuate infection without affecting bacterial growth, this effectively circumvents the resistance problem [15]. For example, 5-FU is a potent quorum-quencher that prevents adhesion and biofilm development by blocking the production and release of quorum sensor autoinducer-2, thereby re-establishing antibiotic susceptibility against MRSA [16]. Apicidin, a fungal metabolite, counters both resistance and virulence due to its potent inhibition against quorum sensing through antagonizing all MRSA accessory gene regulator (Agr) systems in a non-bactericidal manner [17]. With this knowledge, recent strategies for treating microbial infections mainly focus on suppressing virulence characteristics rather than directly killing bacteria.

Repurposing existing drugs to fight against bacterial infections has a definite advantage, with an existed safety profile and a shortened development period. Recently, numerous studies have reported the anti-biofilm or antimicrobial activity of non-antibiotics, such as bithionol and eltrombopag [18,19]. Visomitin is a derivative of plastoquinone with the C10 hydrophobic linker to the triphenylphosphonium (TPP) cation and was previously developed to sustain and restore mitochondrial function and block apoptosis in mitochondrial conditions. It holds promise for treating ocular surface inflammation like dry eye disease [20–22]. Besides, Wei et al revealed that Visomitin might enhance cell proliferation and migration, thereby promoting corneal epithelial wound healing [21]. The antimicrobial activity of Visomitin has only been reported sporadically against *Bacillus subtilis*, *Rhodococcus fascians, Staphylococcus aureus Rosenbach 1884* and *Mycobacterium tuberculosis* [23–25]. Few studies have explored the anti-quorum sensing efficacy of Visomitin against *S. aureus.*

In the current study, we initially identified the antimicrobial activity and the underlying mode of action of Visomitin against *S. aureus*. Then, we mainly focused on exploring the effects of Visomitin as a quorum-quencher interfering with *S. aureus*. Collectively, these results highlighted the antimicrobial and anti-quorum sensing potential of Visomitin, providing an experimental basis for applying Visomitin to treat MRSA-related refractory infections.

## Results

### Bactericidal activity of visomitin against *S. aureus*

**Figure 1.**
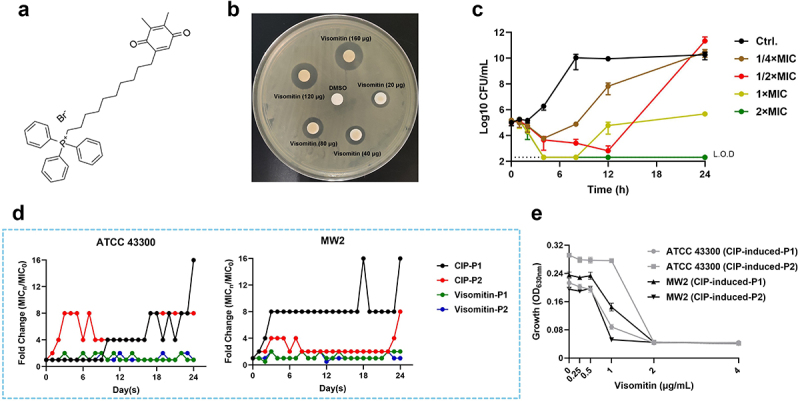
Antimicrobial activity of visomitin against *S. aureus*. (a) The structural formula of visomitin. (b) Disc diffusion assay of visomitin against MRSA ATCC 43,300. The discs contain 20 μg, 40 μg, 80 μg, 120 μg, 160 μg of visomitin, respectively. DMSO (8 μL) was used as a control. (c) Time-dependent killing of visomitin against MRSA ATCC 43,300 at 1/4 - 2 × MIC (0.5 - 4 μg/mL). L.O.D., limit of detection. (d) Resistance development of *S. aureus* ATCC 43,300 and MW2 by visomitin or ciprofloxacin (CIP) at the concentration of 1/2 × MIC (1 μg/mL) in duplicate (P1 and P2). (e) Dose-dependent growth inhibition of visomitin against the cip-induced highly resistant *S. aureus* ATCC 43,300 and MW2.

Visomitin [(10-(4,5-dimethyl-3,6-dioxocyclohexa-1,4-dien-1-yl) decyl) triphenylphosphonium bromide], a peroxidation inhibitor, contains the plastoquinone structure and three phenyl groups as shown in Figure 1a. To explore the antimicrobial potential of Visomitin, the micro-broth dilution assay was performed. As presented in Table 1, Visomitin demonstrated potent bactericidal activity against type strains and clinical isolates including both MRSA and MSSA with the MIC and MBC values of 2 μg/mL and 2–8 μg/mL, respectively. Additionally, Visomitin also exerted strong antimicrobial properties against *S. epidermidis* and *Enterococci*. However, the relatively high MIC values (16–64 μg/mL) indicated weak antimicrobial effects of Visomitin against Gram-negative strains.

**Table 1.** Antibiotic susceptibility of visomitin against pathogens.

Strains	Resistance pattern	Visomitin		DAP^g^	
		MIC (μg/mL)	MBC (μg/mL)	MIC (μg/mL)	MBC (μg/mL)
*S. aureus*					
ATCC 25,923	MSSA^a^	2	4	2	4
ATCC 29,213	MSSA	2	4	2	2
MW2	MSSA	2	4	2	2
USA300	MRSA^b^	2	2	2	4
ATCC 43,300	MRSA	2	2	1	2
LZB1	MSSA	2	8	2	2
RJ-2	MSSA	2	4	1	1
RJ-2 *Δagr*	MSSA	2	4	0.5	1
SA1908	MSSA	2	2	2	4
SA1911	MSSA	2	2	2	4
SA1912	MSSA	2	2	2	8
SA1913	MSSA	2	2	2	4
SA1915	MSSA	2	4	2	4
SA1916	MSSA	2	4	2	2
SA0645	MRSA	2	2	2	2
82174	MRSA	2	8	2	2
82231	MRSA	2	2	2	2
*S. epidermidis*					
RP62A	MSSE^c^	2	2	2	8
ATCC 12,228	MSSE	2	2	1	2
*E. faecalis*					
ATCC 29,212	VSE^d^	2	4	8	8
ATCC 51,299	VRE^e^	4	4	16	16
*E. faecium*					
ATCC 19,434	VSE	4	8	8	16
U101	VRE	2	2	16	32
*E. coli*					
ATCC 25,922	Non-MDR^f^	64	64	-	-
*K. pneumoniae*					
ATCC 700,603	Non-MDR	64	64	-	-
*A. baumannii*					
ATCC 19,606	Non-MDR	16	16	-	-
*P. aeruginosa*					
PAO1	Non-MDR	64	64	-	-

^a^: Methicillin-sensitive *S. aureus*; ^b^: Methicillin-resistant *S. aureus*; ^c^: Methicillin-sensitive *S. epidermidis*; ^d^: Vancomycin-sensitive *enterococcus*; ^e^: Vancomycin-resistant *enterococcus*; ^f^: Non- Multidrug resistance; ^g^: daptomycin.

Subsequently, we conducted a disc diffusion assay and observed a dose-dependent inhibition of MRSA ATCC 43,300 growth by Visomitin (Figure 1b). The bactericidal kinetic study also indicated that Visomitin showed dose- and time-dependent bactericidal activity against MRSA ATCC 43,300. For example, the amount of viable bacterial cells rapidly reduced to the limit of detection after being treated with 2 × MIC of Visomitin for 4 h (Figure 1c). Under resistance induction assay at sub-MICs, we observed a 16-fold increase of the MIC values by CIP over 24 passages, whereas Visomitin remained unchanged throughout the experiment (Figure 1d). In addition, Visomitin still exhibited effective growth inhibitory effects against the CIP-induced strains at 1 × MIC (Table 2), which indicated no cross-resistance between Visomitin and CIP (Figure 1e). Intriguingly, unlike the changes in growth curves (Figure S1A), the variation of morphometric and resistance in Visomitin-induced *S. aureus* MW2 were particularly evident. We observed the inhibited haemolysis with pale colonies appearances on sheep blood agars (Autobio, Zhengzhou, China) by the Visomitin-induced strains (Figure S1B). The Visomitin-induced strains showed significantly decreased sensitivity to erythromycin (E) and clindamycin (DA) compared with the wild type (Figure S1C). The MIC values were presented in Table S1. Meanwhile, we also noticed the altered antimicrobial susceptibility of other conventional antibiotics against Visomitin-induced strains (Figure S1D). The mechanisms underlying the changing tolerance of Visomitin-induced strains to conventional antibiotics were unknown.

**Table 2.** Antimicrobial susceptibility of visomitin against cip-induced *S. aureus.*

Strains	ATCC 43,300^a^		MW2^b^	
	CIP-induced-P1	CIP-induced-P2	CIP-induced-P1	CIP-induced-P2
MIC (μg/mL)	2	2	2	2
MBC (μg/mL)	4	4	4	2

^a^: MRSA type strain; ^b^: MSSA type strain.

In recent years, antimicrobial combinations have been widely used in clinical settings for the treatment of refractory infections. Consequently, we further conducted a chequerboard assay to assess whether Visomitin could exert synergistic antimicrobial effects with conventional antibiotics. However, as shown in Figure S2, no obvious synergies were observed. Visomitin only exhibited additive or irrelevant effects when combined with some antibiotics with the FICIs ranging from 0.75–1.5.

### Bacterial cell membrane disruption by Visomitin

For its rapidly bactericidal activity against *S. aureus*, we presume that Visomitin may function through bacterial cell membrane damage. The scanning electron microscope (SEM) images displayed clustering of bacterial cells with blurred edges and distorted surfaces after Visomitin treatment (Figure S3A). Consistently, the transmission electron microscope (TEM) images showed that there were one or more mesosome-like structures in *S. aureus* cells after treatment with Visomitin, and the cell membrane dissolved, while the edges of untreated cells were clear, without noticeable ultrastructural changes (Figure S3B). The frequently appearing mesosome-like structures may imply a membrane-related stress response against the Visomitin treatment [26]. Through the ultrastructural observation, we initially speculated that Visomitin might exhibit antibacterial activity by disrupting the bacterial cell membrane.

Further, we proceeded to conduct the related membrane permeability assays. SYTOX Green fluorescent dye binds to bacterial nucleic acids through disrupted cell membranes [27]. As we expected, we observed that Visomitin induced a dose-dependent increase in membrane permeability within 30 min, as illustrated in Figure S3C. However, Visomitin did not exhibit more fluorescence increase at the concentration of 4 × MIC, probably indicating the complete lysis of the bacteria. DiSC3(5) is stable in the intact polarized cell membranes, and the fluorescent dye will not be detected until the membrane potential is disrupted [28]. When *S. aureus* was incubated with Visomitin at a concentration over 1 × MIC, strong fluorescence was detected, indicating rapid disruption of the cell membrane by Visomitin (Figure S3D). Overall, it is reasonable to assume that Visomitin targets bacterial cell membranes, which can cause leakage of cellular components and ultimately lead to bacterial mortality.

### Bactericidal activity of Visomitin against *S. aureus* high tolerant cells

**Figure 2.**
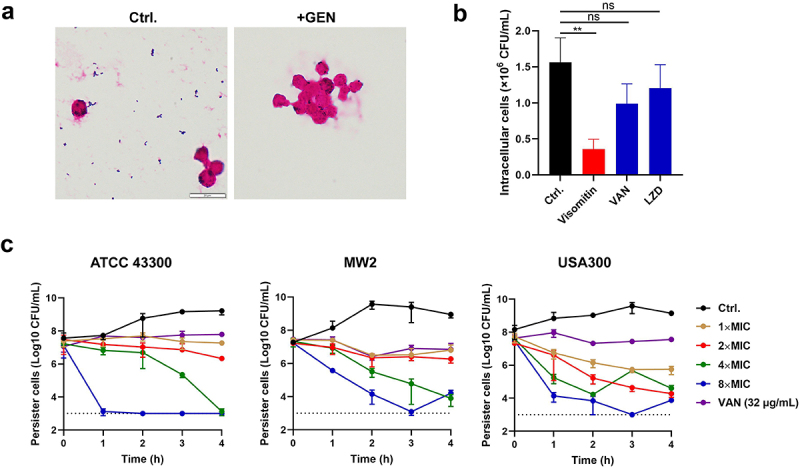
Bactericidal activity of visomitin against *S. aureus* intracellular cells and persister cells. (a) Representative images of MRSA USA300 endocytosed by Raw264.7 observed by gram staining. The extracellular bacteria were eliminated by GEN (100 μg/mL) treatment. Magnification: 400 × . (b) Intracellular killing activity against MRSA USA300 by visomitin. VAN and LZD were used as controls. (c) Bactericidal effects of visomitin against *S. aureus* persister cells of MRSA ATCC 43,300, MSSA MW2, and MRSA USA300. VAN (32 μg/mL) was used as a control.

Considering the tolerant phenotype of *S. aureus*, we further explored the antimicrobial efficacy of Visomitin against the intracellular bacteria and persister cells *in vitro*. The intracellular bactericidal assay was performed using Raw 264.7 cells. MRSA USA300 was engulfed by activated Raw 264.7 cells, and then the extracellular bacteria were killed with high concentrations of GEN (Figure 2a). After 3 h treatment with Visomitin (4 × MIC), the intracellular bacteria were rapidly reduced to less than 0.5 × 10^6^ CFU/mL, suggesting that Visomitin could penetrate the mammal cell membranes and exhibited bactericidal activity against intracellular bacteria. In contrast, vancomycin (VAN) and linezolid (LZD) at 200 μg/mL showed none or weak antimicrobial activity against the intracellular bacteria (Figure 2b). Moreover, Visomitin exhibited rapid, dose- and time-dependent bactericidal activities against MRSA ATCC 43,300 persister cells. The number of surviving bacterial cells reduced to ~ 1 × 10^3^ CFU/mL after incubating with 8 × MIC of Visomitin for 1 h. Similar results were also observed in MSSA MW2 and MRSA USA300 (Figure 2c). Therefore, it could be concluded that Visomitin demonstrated effective antibacterial activities against both *S. aureus* and its high tolerance phenotypes.

### Haemolysis inhibitory activity of Visomitin at sub-MICs

**Figure 3.**
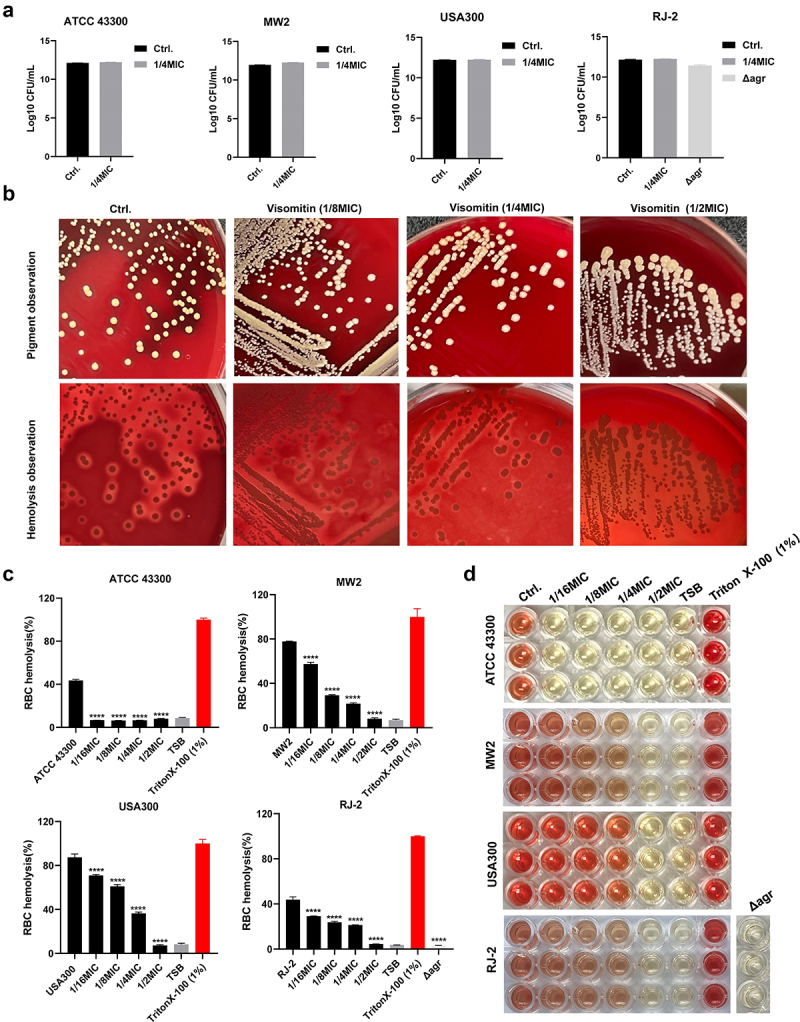
Inhibition of haemolytic activity of *S. aureus* by Visomitin at the sub-MICs. (a) Bacterial count after treatment with Visomitin at the concentration of 1/4 × MIC (0.5 μg/mL). (b) Representative images of dose-dependent inhibition of haemolytic activity against *S. aureus* MW2 by Visomitin on blood plates. (c) Quantitative analysis of haemolytic activity inhibition by Visomitin. (d) Representative images of the haemolysis inhibition in a 96-well cell plate. ****: *p* < 0.0001.

The growth pattern of *S. aureus* ATCC 43,300, MW2, USA300, RJ-2, and RJ-2 *Δagr* were determined to ensure that sub-MICs of Visomitin do not induce any selection pressure on *S. aureus*. No significant change in bacterial density of tested *S. aureus* was observed in the Visomitin-treated (1/4 × MIC) and untreated groups after co-culture for 24 h (Figure 3a). Considering the reduced haemolytic ability of the Visomitin-induced strains in Figure S1B, we subsequently inoculated *S. aureus* MW2 wild-type on the Visomitin-loaded blood agar. As we expected, the sizes of haemolytic rings were dose-dependent, and the haemolytic ring completely disappeared at 1/2 × MIC without any inhibition of colony growth (Figure 3b). Further, we quantified the inhibitory effect of Visomitin at 1/16 - 1/2 × MIC using erythrocyte haemolysis inhibition assay. Consistently, the haemolytic activity of the tested strains was notably inhibited at 1/16 × MIC in a dose-dependent manner (Figure 3c-d). When the erythrocytes incubated with Visomitin-treated *S. aureus* supernatants at 1/16 × MIC and 1/8 × MIC, 6.70–71.06% and 6.34–61.06% of erythrocytes were lysed compared to the 1% Triton X-100 treated (100%), respectively (Figure 3c). For *S. aureus* ATCC 43,300, a significant reduction in lysed erythrocytes was observed after the treatment with Visomitin at 1/16 × MIC − 1/4 × MIC, and the colour of the solution was comparable to that of the TSB-treated group, suggesting the haemolytic activity was entirely suppressed. Compared to *S. aureus* RJ-2 *Δagr*, the RJ-2 wild type exhibited higher haemolytic activity (Figure 3c-d). All these findings implied that the low concentrations of Visomitin could remarkably prevent the haemolytic activity of *S. aureus*.

### Inhibition of staphyloxanthin production by Visomitin

**Figure 4.**
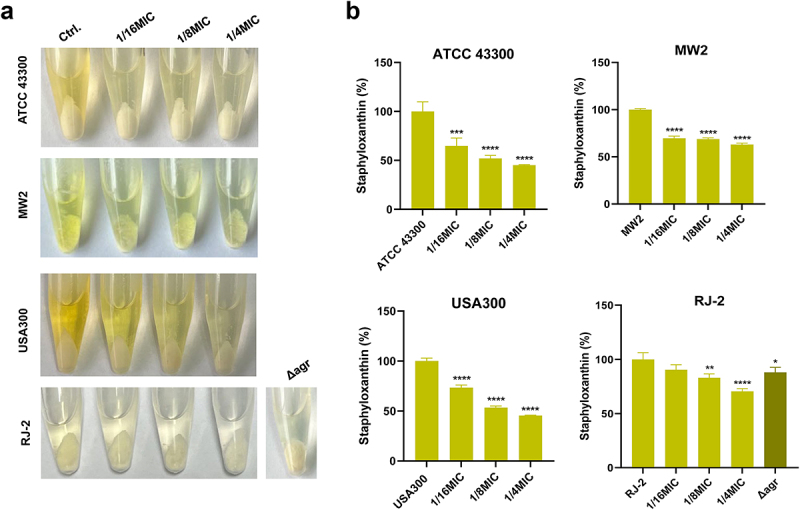
Inhibition of visomitin on staphyloxanthin production. (a) Representative images of pigment extraction using methanol. (b) Qualitative assessment of staphyloxanthin production of *S. aureus* strains after sub-MICs of Visomitin treatment. *: *p* < 0.05; **: *p* < 0.01; ***: *p* < 0.001; ****: *p* < 0.0001.

Although Staphyloxanthin is not essential for growth and reproduction, it involves the pathogenic process of *S. aureus*, and more than 90% of clinical strains produce this pigment [29,30]. We were surprised to discover that Visomitin-induced *S. aureus* MW2 growing on blood agar plates exhibited more pale appearances than the wild type (Figure S1B). To explore whether Visomitin is the inhibitor of staphyloxanthin biosynthesis of *S. aureus*, we incubated *S. aureus* in the absence and presence of Visomitin. Indeed, Visomitin significantly inhibited staphyloxanthin production (Figure 4a-b). After staphyloxanthin extracted using methanol, we observed that the colour of the pigment of Visomitin-treated *S. aureus* changed from golden yellow to colourless (Figure 4a). As presented in Figure 4b, the staphyloxanthin production of *S. aureus* ATCC 43,300, MW2, and USA300 was highly inhibited by 1/16 × MIC of Visomitin with 26.70–35.32%, while the staphyloxanthin production of RJ-2 was suppressed by 1/8 × MIC of Visomitin with 17.08%. In addition, *S. aureus* RJ-2 *Δagr* exhibited weaker pigment production than its wild type. We conclude that Visomitin blocked staphyloxanthin biosynthesis of *S. aureus*, and that the pigment inhibitory action of Visomitin is not limited to a particular strain.

### Inhibition of biofilm formation by Visomitin

**Figure 5.**
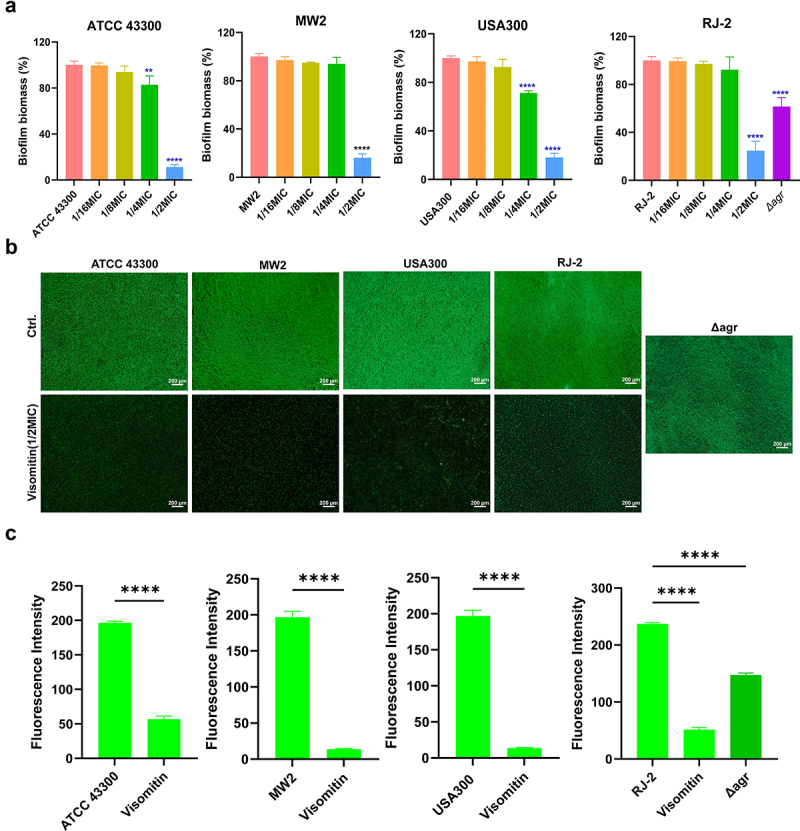
Effects of Visomitin on biofilm formation of *S. aureus*. (a) Dose-dependent biofilm inhibition effect of Visomitin determined by crystal violet staining. (b) Representative fluorescence images of the biofilm inhibition effects of Visomitin by SYTO9/PI staining. (c) Fluorescent quantitative analysis by image J. **: *p* < 0.01; ****: *p* < 0.0001.

By using crystal violet staining, we found that the biofilm of MRSA ATCC 43,300 and USA300 began to be destroyed after treated with Visomitin at the concentration of 1/4 × MIC, and 1/2 × MIC of Visomitin could effectively inhibit the biofilm formation of all the tested strains with a biomass reduction of 75.20–88.81% (Figure 5a). Following SYTO9/PI staining, we observed a reduction in biofilm density with weak green fluorescence, indicating a pronounced biofilm inhibition effect after treatment with Visomitin at 1/2 × MIC (Figure 5b). The quantitative analysis of the fluorescence intensity also verified the antibiofilm activity by Visomitin (Figure 5c). Consistently, the untreated *S. aureus* RJ-2 *Δagr* mutation showed weak biofilm-forming ability compared with the wild type (Figure 5a). Same results were also observed for biofilm fluorescence staining (Figure 5b–c).

### Inhibition of self-aggregation and colony spreading by Visomitin

**Figure 6.**
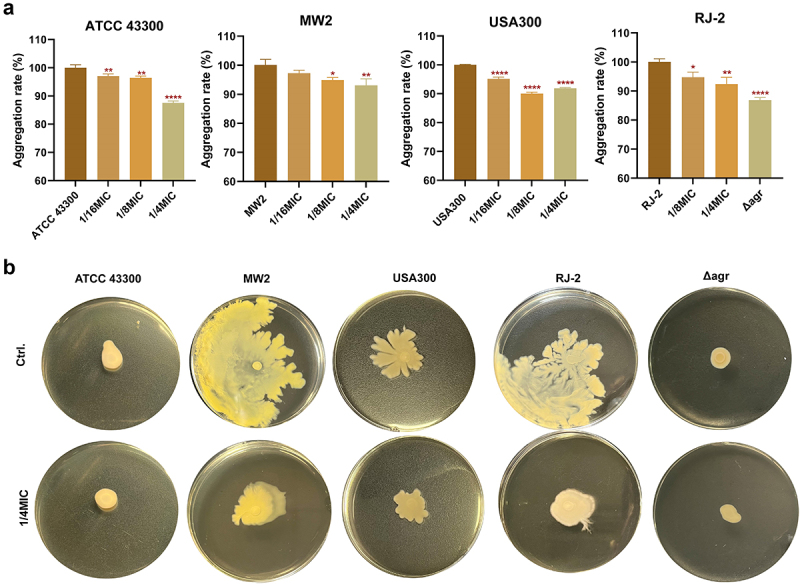
Additional inhibitory effects of visomitin against *S. aureus*. (a) Quantitative analysis of *S. aureus* aggregation after sub-MICs of visomitin treatment. (b) Inhibition of visomitin on colony spreading of *S. aureus*. *: *p* < 0.05; **: *p* < 0.01; ****: *p* < 0.0001.

*S. aureus* settles to form a multicellular cluster without intervention, known as aggregation. Bacterial self-aggregation refers to the mutual adhesion of bacteria, which is closely related to biofilm formation and quorum sensing [31]. Accordingly, we determined whether Visomitin inhibited *S. aureus* aggregation in this study. Although the aggregation inhibition rates of the tested *S. aureus* were various, ranging from 5.29% to 9.96% at the concentration of 1/8 × MIC, the data revealed that the aggregation decreased dose-dependently after treatment with series sub-MICs of Visomitin (Figure 6a).

The motility of *S. aureus* regulated by quorum sensing is also known as colony spreading and has been identified in relation to the pathogenic mechanism of *S. aureus* [32]. In this study, we evaluated the inhibitory action of Visomitin on the colony spreading ability of *S. aureus* and observed a significant decrease at 1/4 × MIC (Figure 6b). Interestingly, we found that the MSSAs of MW2 and RJ-2 exhibited better ability of spreading compared with the MRSAs of ATCC 43,300 and USA300. Additionally, consistent with the idea that the Agr quorum sensing system might facilitate colony spreading, our results showed that RJ-2 *Δagr* hardly spread on soft agar plates.

### Visomitin down-regulated the quorum sensing-related genes expression

**Figure 7.**
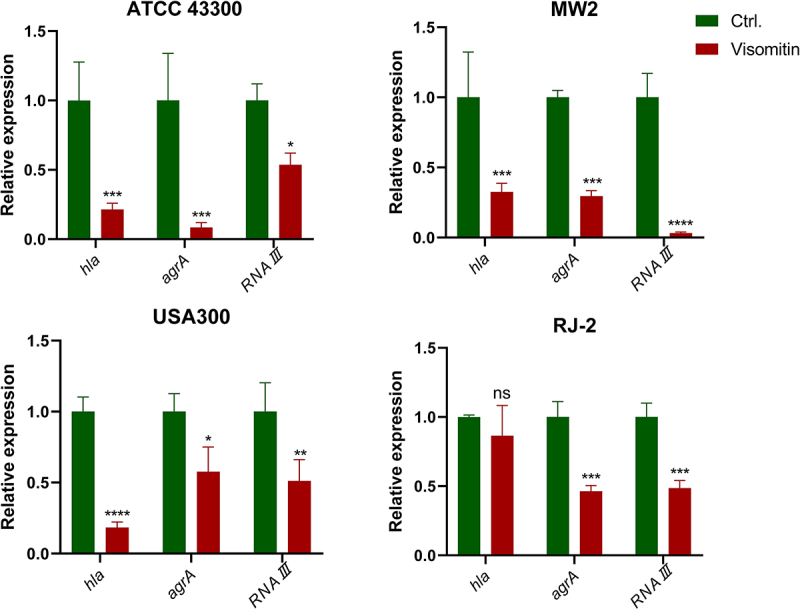
Quantitative expression of genes associated with biofilm and virulence including *hla*, *agrA*, and *RNAIII*. *: *p* < 0.05; **: *p* < 0.01; ***: *p* < 0.001; ****: *p* < 0.0001.

*S. aureus* produces various virulence molecules and metabolites during pathogenesis. The expression of these molecules is mainly regulated by the Agr quorum sensing system, and one of the transcript effectors is RNAIII [33]. To probe the mechanism by which Visomitin inhibited virulence, we compared the RNA levels of haemolysis- and quorum sensing system-related genes in the presence or absence of Visomitin. The results indicated that the expression of *hla*, *agrA*, and *RNAIII* genes was significantly downregulated to varying degrees after Visomitin treatment. However, there was no change in *hla* expression in *S. aureus* RJ-2 (Figure 7).

### Acceptable cytotoxicity of Visomitin

**Figure 8.**
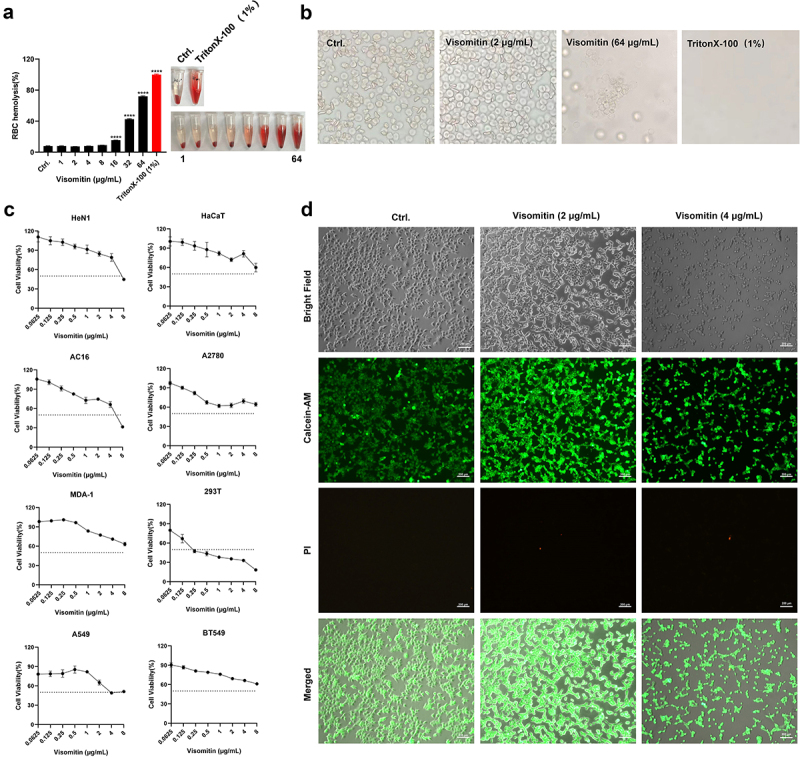
Cytotoxicity determination of Visomitin. (a) Haemolytic activity of Visomitin to human erythrocytes. (b) Representative images of human erythrocytes in the presence or absence of Visomitin. 1% (vol/vol) triton X-100 and 0.1% (vol/vol) DMSO were used as positive and negative controls, respectively. Magnification: 400 × . (c) The cell viability of HeN1, HaCaT, AC16, A2780, MDA-1, HEK293T, A549, and BT549 after treatment with Visomitin for 24 h determined by CCK-8 assay. (d) Cell viability observation of HEK293T by calcein-AM and PI staining. Scale: 20 μm. Green and red fluorescence represent the live and dead cells, respectively. ****: *p* < 0.0001.

Human erythrocyte haemolysis and CCK-8 cytotoxicity assays were conducted to assess the toxicity of Visomitin *in vitro*. We found that Visomitin showed no haemolysis activity up to 8 × MIC (Figure 8a), and the morphology of erythrocytes was kept unchanged in the presence of 1 × MIC of Visomitin by microscopy observation (Figure 8b). In addition, Visomitin exhibited only moderate cytotoxicity to HeN1, HaCaT, AC16, A2780, MDA-1, A549, and BT549 cell lines with the half maximal inhibitory concentration (IC50) of 4–8 μg/mL (higher than the value of MIC). Although higher toxicity was observed in HEK293T cells with IC50 of 0.25 μg/mL (Figure 8c), the dead/live cells detection by Calcein-AM and PI staining showed that Visomitin did not induce HEK293T cell death around the MIC (Figure 8d).

### Antibacterial activity of Visomitin in vivo

**Figure 9.**
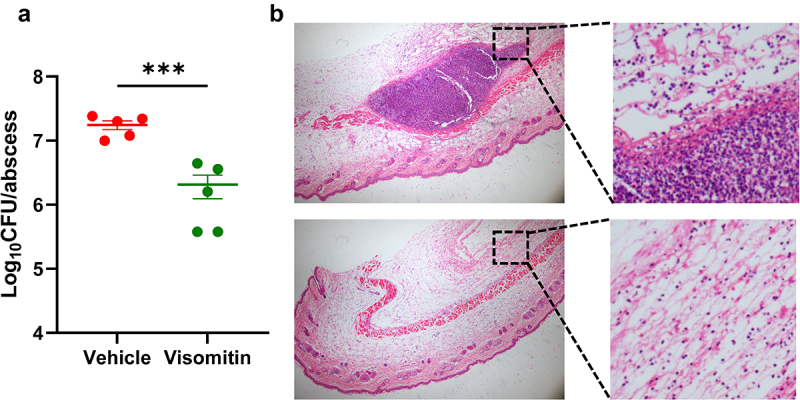
Antibacterial efficacy of visomitin against ATCC 43,300 *in vivo*. (a) The viable bacterial loads of the abscess after the treatment with visomitin (20 mg/kg). (b) Representative images of H&E staining of the skin abscesses treated or untreated with visomitin. ***: *P * < 0.001.

The murine skin abscess model infected by MRSA ATCC 43,300 was used to explore the antimicrobial efficacy of Visomitin *in vivo*. As shown in Figure 9a, a significant bacterial cell loads reduction (0.93-log) was observed after treated with 20 mg/kg of Visomitin compared to the vehicle group. Consistently, H&E staining showed that almost no inflammatory infiltration was observed in the mice treated with 20 mg/kg of Visomitin, while the vehicle group exhibited severe inflammatory infiltration (Figure 9b).

## Discussion

Compounds with TPP, like Visomitin, usually show great affinity towards the membrane by taking advantage of the hydrophobicity and positive charge [34]. Considering the properties of Visomitin, we performed the antibiotic susceptibility tests and found that Visomitin exhibited potent antimicrobial and bactericidal activity against MRSA with a low resistance occurrence rate. Similar bactericidal activity of Visomitin was observed against other Gram-positive coccis like *S. epidermidis* and *Enterococcus sp*. However, it showed weak antibacterial activity against Gram-negative bacteria. It is generally known that Gram-positive bacteria do not form a permeability barrier for small molecules diffusion due to the relatively unitary and porous peptidoglycan layers. In contrast, the peptidoglycan of Gram-negatives was surrounded by an outer membrane containing lipopolysaccharides which blocks the entry of numerous hydrophobic antibiotics. We subsequently conducted electron microscope observation and mechanistic studies using the SYTOX Green permeability assay and DiSC3(5) membrane potential assay. As expected, Visomitin exerted antibacterial efficacy by enhancing cell membrane permeability and disrupting membrane potential which is consistent with the findings of Nazarov et al [25]. We concluded that positively charged Visomitin easily penetrates through bilayer lipid membranes and readily attaches to the cell membranes due to the electrostatic interaction which could disrupt the membrane structure and destroy cell integrity. Current clinical studies suggested that persister cells have been overlooked during *S. aureus* infections, similar to other antibiotic-resistant bacteria, which resulted in antibiotic failure and chronic refractory infections [35,36]. Surprisingly, our findings indicated that Visomitin exhibited more vigorous bactericidal activity against *S. aureus* tolerant phenotypes, including persister cells and intracellular bacteria, compared with high concentrations of VAN. In conclusion, Visomitin, as a cell membrane disruptor, demonstrated irreplaceable properties such as fast killing and powerful bactericidal activity, providing effective therapeutic strategies for clinical use.

Quorum sensing has attracted much attention as a key regulatory hub for the virulence production of *S. aureus*. Most of the virulence factors that are essential for the establishment of infection are regulated by the “accessory gene regulator Agr.” Developing quorum sensing inhibitors by targeting the Agr may be an optimistic therapeutic strategy with a lower incidence of resistance development. In this study, in addition to bactericidal activity, we discovered that sub-MICs of Visomitin could strongly depress quorum sensing-related activities of *S. aureus*. In detail, low concentrations of Visomitin could significantly reduce the haemolytic activity of *S. aureus* which significantly alleviates tissue damage to the host caused by *S. aureus*. Staphyloxanthin, which serves as an oxidant defence system and protects *S. aureus* from host oxidant damage, was inhibited by sub-MIC of Visomitin. It is well known that the extracellular matrix formed by *S. aureus* under diverse growth circumstances along with the maturity and cell concentration of the biofilm, remarkably influenced the efficacy of antibiotics [37,38]. We surprisingly found that Visomitin could reduce biofilm thickness or even inhibit biofilm formation. Bacterial aggregation is the first step of biofilm formation, called initial attachment, while the colony spreading of *S. aureus* facilitates bacterial colonization. Low-dose of Visomitin showed potent inhibition on the aggregation and colony spreading of *S. aureus*. Beyond that, we have found that the transcription of several quorum sensing genes such as *agrA* and *RNA III* were also affected by low concentrations of Visomitin. The Agr quorum sensing system of *S. aureus* consists of two transcriptional units, RNAII and RNAIII, where RNAII contains four genes (*agrA*, *agrB*, *agrC*, and *agrD*). RNAIII is coactivated by AgrA and regulates a series of downstream virulence factors such as *hla* and *pvl* [39,40]. Inhibition of *agrA* transcription will affect the production of AgrA, a response regulator of the two-component signal transduction system, thereby preventing RNA II and RNA III from initiating transcription and further suppressing virulence production. Decreased transcription of *RNA III* and *hla* could also support this view. Different from conventional antibiotics, sub-MICs of Visomitin could interfere with quorum sensing, which does not aim to kill the pathogens but minimizes the risk of resistance development.

Visomitin, with its dual antimicrobial actions of anti-virulence at low concentration and bactericidal action at high concentration, is undoubtedly a desirable antimicrobial agent in clinical settings. Nevertheless, one of the major obstacles concerning practical applications of Visomitin could be pro-oxidant at high concentrations. Decreased cell viability and metabolic activity following the treatment of Visomitin above 1 μM were observed by Carlos Fernandes et al [41]. Although mammalian cells survived more than 50% at effective antimicrobial concentrations of Visomitin, it still shows certain cytotoxicity (Figure 8). Considering that antibiotics combination might decrease the dose of Visomitin to reduce its toxicity, we demonstrated drug combination test and the results suggest that Visomitin does not show synergistic effects in combination with antibiotics. Thus, we explored the bioactivity of Visomitin below the MICs which could achieve the anti-virulence activity with satisfied safety.

Besides, some potential limitations need to be clarified in our study, and further exploration is still needed to facilitate its clinical application. Although long-term use of Visomitin did not induce *S. aureus* resistance to Visomitin, the induced strain exhibited resistance to other classes of antibiotics such as E and DA. It suggests that Visomitin does not have a similar mechanism of action to that of macrolides or lincomycins, on the other hand, cross-resistance needs to be paid attention to. Despite we have concluded that Visomitin reduced bacterial loads and attenuated skin damage in the skin abscess model *in vivo*, additional experiments, such as the pneumonia model or bacteraemia model, are still needed to thoroughly assess the antimicrobial efficacy of Visomitin *in vivo*. Furthermore, although we have demonstrated that the transcriptions of *agrA* and *RNA III* were influenced by low concentrations of Visomitin, Visomitin interacted with the Agr system is still unknown.

## Conclusion

In summary, this study reported that Visomitin demonstrated desirable antimicrobial activity against *S. aureus in vitro* and *in vivo*. Sub-MICs of Visomitin significantly reduce virulence production and biofilm formation in *S. aureus* by interfering with the transcription of Agr quorum sensing genes. These findings provided new evidence for developing Visomitin as a novel antibacterial agent or quorum-sensing inhibitor to overcome refractory *S. aureus* infections.

## Materials and methods

### Strains, culture conditions, and chemicals

All the type strains, including *S. aureus* ATCC 43,300, *S. aureus* USA300, *S. aureus* MW2, *S. aureus* ATCC 25,923, *S. aureus* ATCC 29,213, *S. aureus* RJ-2, *S. epidermidis* RP62A, *S. epidermidis* ATCC 12,228, *Enterococcus faecalis* ATCC 29,212, *Enterococcus faecalis* ATCC 51,299, *Enterococcus faecium* ATCC 19,434, *Escherichia coli* ATCC 25,922 and *Klebsiella pneumoniae* ATCC 700,603 were purchased from the American Type Culture Collection (ATCC). *Pseudomonas aeruginosa* PAO1 was given by Qiao Minqiang (College of Life Sciences of Nankai University, Tianjin, China). Clinical strains of *S. aureus* were collected from the Third Xiangya Hospital of Central South University and identified by VITEK 2 Compact (bioMerieux, France) and Matrix-Assisted Laser Desorption Ionization (BD, Germany). All cell lines were cultivated in DMEM or 1640 (KeyGEN Biotech, China) containing 10% Fetal Bovine Serum (FBS) (Biological Industries, Israel). Visomitin, gentamycin (GEN), vancomycin (VAN), daptomycin (DAP), ciprofloxacin (CIP), linezolid (LZD), and other antimicrobials were purchased from MedChem Express (New Jersey, United States) and dissolved in either dimethyl sulphoxide (DMSO, Sigma-Aldrich, Shanghai, China) or ddH_2_O (TansGen Biotech, Beijing, China).

### Antimicrobial susceptibility test

According to the Clinical Laboratory Standards Institute, the microdilution method was used to determine the minimum inhibitory concentration (MIC) [42]. In brief, fresh bacterial colonies of all strains mentioned above were picked from the sheep blood plate, adjusted to 0.5 McFarland (McF) in saline, and then diluted 1:200 with Mueller – Hinton broth (MH, Solarbio, China). Subsequently, 50 μL of two-fold diluted antimicrobials and 50 μL of the prepared bacterial suspension were transferred to a 96-well plate. After culturing at 37°C for 16–18 h, the MIC was defined as the lowest concentration that prevented visible bacterial growth. For minimum bactericidal concentration (MBC) determination, 5 μL of the bacterial suspension at or above the concentration of MIC was spotted on the sheep blood plates and incubated at 37°C for 24 h. The MBC was identified as the lowest concentration with no bacterial colony growth.

### Kirby-Bauer Method

The fresh bacterial colonies of *S. aureus* ATCC 43,300 were picked and adjusted to 0.5 McF in saline and then evenly spread on MH agar plates with a swab. After drying at room temperature, 5 mm diameter empty discs were placed on the plates, and antimicrobial storage solutions (20 mg/mL) were added to the empty discs after levelling the volume. Disc loaded with an equal volume of DMSO was used as a control. The diameters of the inhibition zones were measured after incubation at 37°C for 18–24 h [43].

### Time-killing assay

The overnight cultured *S. aureus* ATCC 43,300 suspension was diluted 1:200 into 15 mL with MH broth and co-cultured with the indicated concentrations of Visomitin in 50 mL centrifuge tubes. At each time point (0, 1, 2, 4, 8, 12, and 24 h), 5 μL of the bacterial suspension serially diluted with saline were spotted on the sheep blood plates for CFU counting [44].

### Resistance induction assay

The MIC values of MRSA ATCC 43,300 and MSSA MW2 were determined as described in the Antimicrobial Susceptibility Test. The bacterial suspension from the 1/2 × MIC was further 1: 1000 diluted with MH broth and complemented with an equal volume of serially diluted antimicrobials to detect the MIC for the following day. The MIC was serially detected for 24 days, and the changes in the values of MIC were analysed by comparison to the initial MIC [42,45]. Two biological repetitions were performed, and CIP was used as a positive control. Visomitin-P1, Visomitin-P2, CIP-P1 and CIP-P2 used in subsequent experiments were obtained from this experiment.

### Scanning electron microscopy (SEM) and transmission electron microscopy (TEM)

The overnight cultured *S. aureus* was sub-cultured in trypticase soy broth (TSB, Solarbio, China) for 2–4 h to logarithmic growth phase and then exposed to 10 × MIC of Visomitin for 1 h. Next, the bacterial suspension was centrifuged at 8000 × g for 2 min and washed twice with 1 × phosphate buffer (PBS, keyGEN, China). After immersing in an electron microscope fixative overnight, the samples were sent for SEM (Hitachi, Tokyo, Japan) and TEM (Hitachi, Tokyo, Japan) observation. 0.1% (vol/vol) DMSO was used as a control [46].

### Cell membrane permeability determination by SYTOX green and DiSC3(5) staining

Overnight cultured *S. aureus* was sub-cultured with TSB broth for 2–4 h to logarithmic growth period. Then, the bacterial suspension was diluted with 5 mm 4- (2-hydroxyethyl) piperazine-1-ethanesulfonic acid (HEPES, Biosharp, China) solution to the OD_630 nm_ of 0.05 supplemented with or without the specified concentrations of Visomitin. SYTOX Green fluorescence (Thermo Fisher Scientific, United States) dye was added to a final concentration of 2 μM. The fluorescence intensity was detected every 2 min for a total of 30 min using a fluorescence microplate reader at the excitation/emission wavelength of 485/525 nm.

For DiSC3(5) staining, the diluted bacterial solution containing varied concentrations of Visomitin was incubated with 2 μM of DiSC3(5) (AAT Bioquest, USA) supplemented with 5 mm glucose and 100 mm KCl for 1 h at room temperature in darkness. Then, the fluorescence intensity was captured every 1 min for a total of 20 min at the excitation/emission wavelength of 622/670 nm. Melittin (10 μg/mL) and 0.1% (vol/vol) DMSO served as positive and negative controls, respectively [47].

### Intracellular bactericidal assay

Raw 264.7 cells were cultured and harvested in 10% serum of 1640 medium to ~ 3 × 10^6^ cells/mL, while overnight cultured *S. aureus* USA300 was diluted to 0.5 McF (1.5 × 10^8^ CFU/mL). Fifty microlitres of Raw 264.7 cells co-incubated with an equal volume of the bacterial suspension at 37°C for 35 min (Raw 264.7/USA300 = 1/50). Then, GEN (50 μg/mL) was used to kill extracellular bacteria. After incubation at 37°C for 80 min, the sample was centrifuged, washed with 2% DMEM, and treated with 100 μL of Visomitin (4 × MIC) at 37°C for 3 h. Subsequently, the treated precipitate was added with 100 μL of 0.2% (vol/vol) Triton X-100, incubated at 37°C for 20 min with shaking, and then 5 μL of the bacterial suspension was 5-fold gradient diluted and spotted on a sheep blood agar for CFU counting. Before and after GEN treatment, the samples were performed with Gram-staining to ensure that all extracellular bacteria were killed. VAN and LZD were used as controls [48]. *S. aureus* USA300 was chosen for this experiment because GEN (50 μg/mL) can completely remove extracellular *S. aureus* USA300 compared to *S. aureus* ATCC 43,300.

### Persister cells killing assay

*S. aureus* ATCC 43,300, MW2, and USA300 were grown in TSB broth at 37°C 200 rpm for 24 h to the platform stage. Subsequently, the bacteria were mixed with indicated concentrations of antimicrobials in 50 mL centrifuge tubes to the final concentration of ~ 1 × 10^8^ CFU/ml. At the time point of 0, 1, 2, 3, and 4 h, 5 μL of the bacterial suspension was harvested and diluted for CFU counting [49].

### Chequerboard assay

The broth microdilution chequerboard test was used to evaluate the combinational antimicrobial efficacy of Visomitin with conventional antibiotics against *S. aureus* ATCC 43,300. Briefly, twofold serial dilutions of Visomitin (0.125–8 μg/mL) or antibiotics were prepared in MH medium and added to the horizontal and vertical rows of the 96-well plate to create an “8 × 8” matrix. The final concentration of the bacterial culture in each well is approximately 5 × 10^5^ CFU. After incubation at 37°C for 16–18 h, the absorbance was measured at 630 nm using a microplate reader. Fractional inhibitory concentration index (FICI) was calculated as follows:

$$\;\;\;\;\;\;\;\;\;\;\;\;\;\;\mathrm{FICI}=\frac{MIC_{A}\left(\mathrm{combination}\right)}{\mathrm{MI}C_{A}\left(\mathrm{alone}\right)}+\frac{MIC_{B}\left(\mathrm{combination}\right)}{\mathrm{MI}C_{B}\left(\mathrm{alone}\right)}$$

MIC_A_ represents the MIC of Visomitin. MIC_B_ represents the MIC-tested antibiotics. Based on the standard criteria of the CLSI guidelines, synergistic effect was defined as FICI ≤ 0.5; additive effect was defined as 0.5 < FICI ≤ 1; indifference effect was defined as 1 < FICI ≤ 4; antagonism effect was defined as FICI > 4.

### Haemolysis activity determination with antimicrobial-loaded human blood agar

Human red blood cells (RBCs) were obtained from Hemo Pharmaceutical & Biological Co. (Shanghai, China). Antimicrobial-loaded blood plate contains MH broth powder with agar at a ratio of 1: 2 and 10% (vol/vol) of human erythrocytes with or without indicated concentrations of Visomitin. After sufficient solidification, the plates were inoculated with *S. aureus* MW2, a highly haemolytic strain, and incubated at 37°C for 24 h. The size of the haemolytic ring was recorded.

### Erythrocyte haemolysis inhibition assay

The overnight culture of *S. aureus* was resuspended 1: 200 with TSB broth and further exposed to different concentrations (1/16 × MIC-1/2 × MIC) of Visomitin at 37°C 200 rpm for 24 h. After centrifugation at 3000 × g for 10 min, the culture supernatant was harvested, mixed with 25 μL human erythrocytes, and then incubated at 37 °C for 30–60 min. The absorbance of the supernatant was recorded at 570 nm following centrifugation at 3000 × g for 5 min. The haemolysis rate was calculated as follows:

$$\mathrm{Hemolysis}\left(\%\right)=\frac{A_{\mathrm{sample}}-A_{\mathrm{TSB}}}{A_{1\%\mathrm{TritonX}-100}-A_{\mathrm{TSB}}}\times 100\%$$

A_TSB_ and A_1%TrintonX-100_ represent the absorbances of the suspension of untreated and treated with 1% (vol/vol) Triton X-100, used as positive and negative controls, respectively [50].

### Measurement of staphyloxanthin production

*S. aureus* bacteria were grown in TSB (10 mL) medium (with or without Visomitin) at 37°C with shaking at 200 rpm for 24 h. Before the assays, bacteria cultures were centrifuged and washed twice with 1× PBS. Then, the pigment was extracted using 800 μL of methanol, followed by boiling for 10 minutes at 100°C and determination of the absorbance values at 450 nm [51]. The staphyloxanthin production rate was calculated as follows:

$$\mathrm{Staphyloxanthin}\left(\%\right)\;=\frac{A_{\mathrm{sample}}-A_{\mathrm{blank}}}{A_{\mathrm{untreated}}-A_{\mathrm{blank}}}\times 100\%$$

A*_blank_* represents the absorbances of methanol.

### Biofilm inhibition assay

One hundred microlitres of Visomitin at double concentrations were added to a 96-well plate with an equal volume of overnight cultured bacterial suspension (initial inoculum of 10^6^ CFU/well) and cultured at 37°C for 24 h. Visomitin works at concentrations from 1/16 × MIC to 1/2 × MIC (0.125–1 μg/mL). The biofilm was stained with crystal violet for 5 min at room temperature (~25°C). After washing 2–3 times with saline, the biofilm was quantified by detecting the absorbance at 570 nm [49]. The results are expressed as a percentage of biofilm mass compared to the untreated group (100%).

### Biofilm staining by SYTO9 and PI

To visualize the influence of Visomitin against biofilm formation, *S. aureus*, at exponential phase, was cultured in TSB broth containing 2% glucose and 2 × MIC of Visomitin. After incubation at 37°C for 24 h, the media was discarded, and the plate was washed twice with 1× PBS to ensure the complete removal of planktonic cells. The biofilms were stained with SYTO9 and PI staining (10 μM) for 15 min at room temperature in darkness. After washing and drying, the biofilm was observed using fluorescence microscopy at the excitation/emission wavelength of 485/498 nm for SYTO9 and 535/617 nm for PI, respectively [52].

### Cell aggregation assay

Cell aggregation was performed as previously mentioned [53]. Briefly, overnight cultured *S. aureus* was added to 10 ml TSB at 1:200 and sub-cultured with the indicated concentration of Visomitin at 37°C and 200 rpm for 24 h. Then, the bacteria cultures were centrifugated at 16,000 × g for 2 min, washed 3 times with 1×PBS, and resuspended with 3 mL 1× PBS in a clean glass tube. Subsequently, the bacterial suspension was determined at 595 nm as the initial OD and left at room temperature. After 24 h, the supernatant was discarded, and the precipitated bacteria was resuspended with 3 mL 1 × PBS. The final OD was subsequently recorded, and the percentage of cell aggregation was calculated as follows:

$$\mathrm{Aggregation}\left(\%\right)=\frac{A_{\mathrm{final}}-A_{\mathrm{blank}}}{A_{\mathrm{initial}}-A_{\mathrm{blank}}}\times 100\%$$

A*_initial_* and A*_final_* indicate the absorbances of the suspension before and after aggregation. A*_blank_* represents the absorbances of PBS. The relative aggregation of untreated controls served as a percentage of the value for 100%.

### Colony spreading assay

The spreading ability was assayed on TSB plates containing 0.24% (wt/vol) agar and indicated concentrations of Visomitin. Two microlitres of overnight cultured *S. aureus* (OD600 of 0.5) were spotted at the centre of the plates and incubated for 24 h at 37°C [54].

### Rt-qPCR analysis

RT-qPCR was applied to analyse the actions of Visomitin on the transcription of genes related to biofilm development and virulence. Overnight cultures of *S. aureus* were 1:200 diluted with TSB in the absence and presence of Visomitin (1/8 × MIC) and inoculated in a 50 mL sterile tube for 24 h. The total RNA was extracted using TRIZOL Reagent (Omega, US) as described previously [55]. cDNA was prepared through reverse transcription using the HiScript II 1st Strand cDNA Synthesis Kit (Vazyme, China), and qPCR was conducted using AceQ qPCR SYBR Green Master Mix (Vazyme, China). RT-qPCR was performed with an initial incubation at 95°C for 2 min, followed by 40 cycles of incubation at 95°C for 30 s, 55°C for 20 s and 72°C for 30 s. 16 sRNA was used as housekeeping genes to normalize the transcript levels of the genes. The threshold cycle (Ct) numbers were determined and analysed using the 2−ΔΔCt approach. The gene-specific primers are presented in Table S2.

### Erythrocyte haemolysis assay

The RBCs were centrifuged at 3000 × g for 10 min, washed three times, and then diluted to 5% (vol/vol) erythrocyte suspension with saline. An equal volume of erythrocyte suspension with indicated concentrations of Visomitin was added to a centrifuge tube and incubated at 37°C for 1 h. After centrifugation at 10,000 × g for 5 min, the absorbance of the supernatant was recorded at 570 nm. The haemolysis rate was determined as before described. Additionally, the morphology of the erythrocyte was observed via microscopy [56].

### Cytotoxicity detection by CCK-8

Cell lines of HEK293T, HeN1, HaCaT, MDA-1, AC16, A2780, A549, and BT549 were cultured overnight in DMEM or 1640 medium containing 10% FBS to 50% confluence. Then, two-fold diluted Visomitin and 0.1% (vol/vol) DMSO (negative control) were added to the culture plate. After incubation at 37°C for 24 h, the cytotoxicity assay was performed using the CCK-8 reagent. In brief, 10 μL of the CCK-8 solution was added to each well and incubated at 37°C for 1 h. The absorbance at 450 nm was subsequently recorded [57]. The cell viability rate was calculated as follows:

$$\mathrm{CellViability}\left(\%\right)=\frac{A_{\mathrm{sample}}}{A_{0.1\%\mathrm{DMSO}}}\times 100\%$$

### Cell viabilities detection by Calcein-AM/PI staining

HEK293T cells were cultured in DMEM containing 10% serum to 50% confluence and then incubated with or without indicated concentrations of Visomitin at 37°C for 24 h. Subsequently, the cells were stained with 2 μM of Calcein-AM and 10 μM of PI for 30 min at room temperature in darkness and observed under fluorescence microscopy at the excitation/emission wavelength of 494/514 nm for Calcein-AM and 493/636 nm for PI, respectively [58].

### Murine subcutaneous abscess model

This study followed the ARRIVE guidelines. All animal experiments were approved by the Ethics Committee of the Third Xiangya Hospital of Central South University (No. 2021sydw0245). Female ICR mice aged 7 weeks weighing approximately 25 ± 3 g were purchased from SAJ Laboratory Animals Ltd (Changsha, China). To ensure the accuracy of the experimental results and to comply with the 3 R principle, mice (*n* = 6 per group) were anesthetized with sodium pentobarbital (50 mg/kg) by intraperitoneal injection. Then, the backs were shaved with an animal electric razor and 100 μL of bacterial suspension containing 10^8^ CFU/mL was subcutaneously injected into the dorsum. Mice were randomly assigned with odd numbers as experimental group and even numbers as vehicle group. At 1 h post-infection, Visomitin (20 mg/kg) was administered via subcutaneous injection. The mice injected with 1% DMSO were used as the vehicle group. After 24 h of infection, the mice were sacrificed, and the skin abscesses were excised for CFU counting and haematoxylin and eosin (H&E) staining, respectively [59].

### Statistical analysis

Unless otherwise mentioned, all experiments were repeated three times separately. Data are generally presented as mean ± standard deviation (SD) using Graph Pad Prism 8.0 software. The distribution normality of all the data was assessed using a QQ plot. The homogeneity of variance was tested with the Brown-Forsythe test. Unpaired Student’s t-test was used to compare the two groups, while one-way ANOVA was used to calculate p-values among multiple groups. Statistical significance was defined as *p* < 0.05 (*p < 0.05, **p < 0.01, ***p < 0.001, ****p < 0.0001).

## Author contribution

RW and YW contributed equally to this study. YW and PS conceived and designed the research. RW and PS conducted most experiments and wrote this manuscript. PW and HL prepared study materials, reagents, or analytical tools. YW, PS, and RW analysed data and made figures. YW and PS reviewed and edited the manuscript. All authors read and approved the manuscript.

## Supplementary Material

Figure_S2.jpg

Figure_S3.jpg

Author Checklist E10 only.pdf

Supplementary Tables.docx

Figure_S1.jpg

## Data Availability

The data that support the findings of this study are directly available in 4TU.ResearchData at https://data.4tu.nl/private_datasets/KqEfoVkjPf6V4F99gH1mxZ4fB5TYKjOHRCnRYfMencI
