# Supplementary figures and images for: Bactericidal and anti-quorum sensing activity of repurposing drug Visomitin against Staphylococcus aureus
Source: Virulence. 2024 Oct 10;15(1):2415952. doi: 10.1080/21505594.2024.2415952 (PMC11492638; doi:10.1080/21505594.2024.2415952)

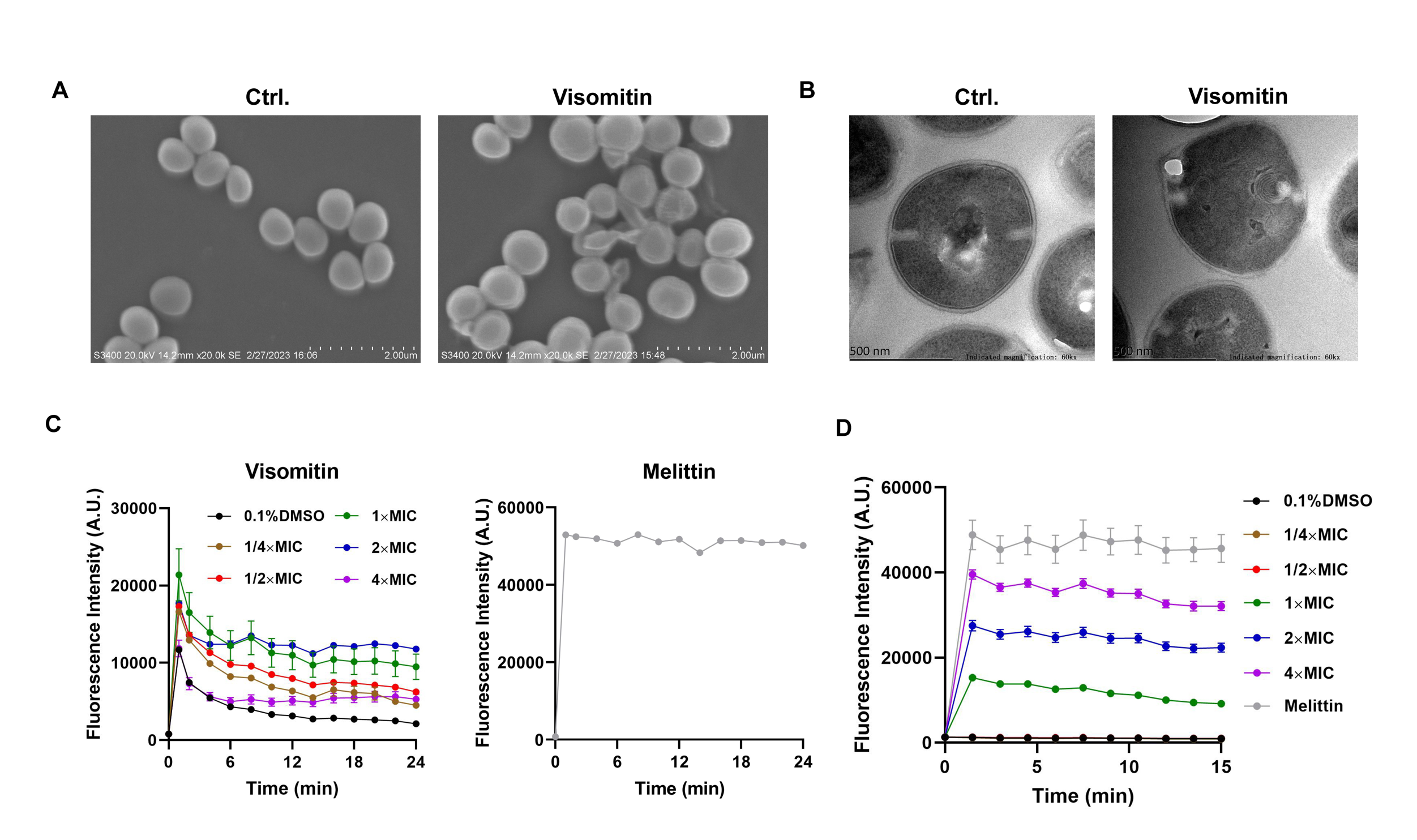

Supplement: Figure_S3.jpg [file KVIR_A_2415952_SM2174.jpg]
