# Supplementary material for: Bactericidal and anti-quorum sensing activity of repurposing drug Visomitin against Staphylococcus aureus
Source: Virulence. 2024 Oct 10;15(1):2415952. doi: 10.1080/21505594.2024.2415952 (PMC11492638; doi:10.1080/21505594.2024.2415952)
Supplement: Supplementary Tables.docx [file KVIR_A_2415952_SM2172.docx]

**Table S1** MICs (μg/mL) of E and DA against MW2 and its Visomitin-induced strains

| Strains | E^a^ | DA^b^ |
| --- | --- | --- |
| WT | 0.25 | 0.0625 |
| Visomitin-induced-P1 | >256 | >256 |
| Visomitin-induced-P2 | >256 | >256 |

^a^: erythromycin; ^b^: clindamycin.

**Table S2** Primers used for RT-qPCR analysis.

| Primer name | Sequences (5′–3′) |
| --- | --- |
| *16s*RNA-F | TGATCCTGGCTCAGGATGA |
| *16s*RNA-R | TTCGCTCGACTTGCATGTA |
| *hla*-F | GCAAATGTTTCGATTGGTCA |
| *hla*-R | CCATATACCGGGTTCCAAGA |
| *agrA-*F | CGAAGACGATCCAAAACAAAG |
| *agrA-*R | ATGTTACCAACTGGGTCATGC |
| *RNAⅢ-*F | AATTAGCAAGTGAGTAACATTTGCTAGT |
| *RNAⅢ-*R | GATGTTGTTTACGATAGCTTACATGC |
